# Prevention of Gas Pains

**Published:** 1919-01

**Authors:** 


***Prevention of Gas Pains.*** By L. A. Emge, *Journal A. M. A.*, Sept. 14, 1918.

The author says that in spite of the progress that has been made in recent years in the rejection of ancient traditions that have clogged the progress of medical science, there is yet a tendency to put some of these traditions above new ideas. One of the best examples of this is the old custom of purging and dieting, in other words, " carefully preparing " patients before operation. The underlying idea of this habit developed in the dark ages of medicine, and in spite of voices of protest that have been raised against it, it still has its status in the profession. Many of these protests are only incidentally mentioned in articles on other subjects, and consequently are not easy of reference. A few of them are mentioned, and the author quotes the evidence afforded by emergency operations. Nobody will deny, Emge says, that a woman with acute appendicitis or tubal pregnancy, or cesarean section properly performed, will recover with little suffering except that caused by the direct wounds, while others " carefully prepared " will often be troubled with most distressing gas pains and distention. While there has been a decided tendency to moderate the purging, judging from recent books and articles, the custom of purging seems to have as strong a footing as it ever had. We now have, however, the results of experiments on animals, some of which are quoted. The essential thing now is to get careful reports of a large, unselected series of purged and nonpurged cases.

Emge's own stimulus for personal observation was received in 1915 in the observation of a number of unselected cases in the Women's Clinic of the University of California Medical School. Since joining the staff of the Women's Clinic of the Leland Stanford Junior University he has carefully recorded the severity of the pains experienced by patients after a hundred major operations. In most of these cases the peritoneum was opened and much pelvic work done. The first fifty were prepared in the old way. The next fifty received no purgation, and the contrast is striking as shown by the tabulated statement. The severe gas pains were observed in 22 per cent. of the purged patients, and in only 2 per cent. of the unpurged ones. Mild gas pains were also similarly infrequent in the latter, and more than double the proportion had no gas pains whatever. While these observations were being made, it was observed that hernia patients suffered often with severe gas pains, and in unpurged cases these were rare or very mild. It is not within the scope of the paper, Emge says, to discuss the treatment of gas pains. Any treatment used should tend to restore normal peristalsis. The best stimulus for this is food, and the earlier it is given the better. Emge summarizes his views as follows: "1. Clinical and experimental observations strongly suggest that preparative purging is a strong factor in the producing of gas pains. 2. At operation the strongly purged bowel is more difficult to handle than the unpurged bowel on acount of congestion and distention. 3. An enema will clear the lower bowel sufficiently for any operative work. 4. Patients who have not been purged are comparatively free from gas pains."

